# Loss of pulmonary tissue protection and neutrophil microbicidal defects promote severe *Aspergillus fumigatus* infection during influenza A virus infection

**DOI:** 10.1128/iai.00234-25

**Published:** 2025-08-11

**Authors:** Zhihan Wang, Taylor Schmit, Kai Guo, Jitendra Kumar Tripathi, Zahrasadat Navaeiseddighi, Antariksh Tyagi, Ramkumar Mathur, Junguk Hur, Donald Jurivich, Nadeem Khan

**Affiliations:** 1 Department of Biomedical Sciences, School of Medicine and Health Sciences, University of North Dakota 710598, Grand Forks, North Dakota, USA; 2 West China School of Basic Medical Sciences & Forensic Medicine, Sichuan University 12530, Chengdu, Sichuan, China; 3 Department of Oral Biology, College of Dentistry, University of Florida 164889, Gainesville, Florida, USA; 4 Department of Neurology, University of Michigan 198418, Ann Arbor, Michigan, USA; 5 Department of Geriatrics, School of Medicine and Health Sciences, University of North Dakota 710859, Grand Forks, North Dakota, USA; University of California Davis, Davis, California, USA

**Keywords:** *Aspergillus fumigatus*, invasive pulmonary aspergillosis, Influenza A Virus, IAV-*Af* co-infection, neutrophils

## Abstract

Invasive pulmonary aspergillosis (IPA) is a severe fungal disease caused by *Aspergillus fumigatus* (*Af*) that may spread hematogenously to extrapulmonary organs. IPA is typically associated with a broad spectrum of immunocompromised conditions and constitutes a high mortality rate. While the association of influenza as a risk for secondary bacterial infections is well appreciated, emerging evidence indicates that influenza-hospitalized patients demonstrate increased susceptibility to severe aspergillosis infection. In this study, we developed a murine Influenza A Virus (IAV)-*Af* co-infection model and investigated the role of IAV host response in promoting invasive *Af* infection. Our data show that IAV temporarily suppresses neutrophil recruitment in the early phase of *Af* co-infection (24 hours), followed by a subsequent increase in neutrophil levels (48 hours). RNA sequencing analysis of neutrophils from IAV-*Af* co-infected lungs (48 hours) reveals enrichment of pathways regulating inflammatory responses and phagocytosis. Despite higher inflammatory response and phagocytosis, the host response from IAV-*Af* co-infected lungs had suppressive effects on neutrophil conidial killing, correlating with lung fungal load and invasion. However, the increased fungal invasion observed at 24 hours post co-infection, despite similar fungal loads in both groups (*Af* vs. IAV-*Af*), suggests that IAV-induced pathologic lung inflammation and vascular damage likely promote *Af* invasiveness during the initial phase of co-infection, and subsequently, the defects in neutrophil fungicidal response and exacerbated lung damage lead to sustained and fatal IPA pathogenesis in the later phase of co-infection.

## INTRODUCTION

Invasive pulmonary aspergillosis (IPA) is a severe fungal disease caused by *Aspergillus fumigatus* (*Af*) (1). IPA is typically associated with a broad spectrum of immunocompromised conditions and immunologic disorders, leading to a high mortality rate (2). The emerging association of influenza virus with severe pulmonary aspergillosis is increasingly recognized (3, 4). Co-infection with *Af* has been reported in approximately 16–28% of influenza-hospitalized patients who required intensive care unit (ICU) admission (5). Furthermore, severe fungal infections in hospitalized patients with COVID-19 highlight the significant role of pulmonary viruses as substantial risks for secondary infections beyond bacteria (6).

IPA pathogenesis primarily involves two key aspects: (1) a weakened phagocytic response that compromises host defense, allowing conidia to germinate into hyphae within the lungs, and (2) hyphal invasion of lung endothelial cells, leading to hematogenous spread to other organs (7). While the role of a compromised phagocytic response as a risk for pulmonary aspergillosis is well recognized, the host factors implicated in influenza-associated pulmonary *Af* infection are not fully elucidated. Further, the relative contribution of phagocytic dysfunction and tissue damage in influenza-associated IPA remains incompletely understood. The limited robustness of available Influenza A Virus (IAV-*Af* co-infection models, compared to influenza-bacterial co-infection models, remains a significant bottleneck in advancing research on the association between IAV and secondary *Af* infections.

We developed a murine IAV-*Af* co-infection model to investigate the association of IAV with severe pulmonary aspergillosis. Our findings show that IAV preferentially enhances the lung host response, dominated by inflammatory monocytes and macrophages, while limiting neutrophil recruitment during the initial phase of IAV-*Af* co-infection (24 hours). However, this impaired neutrophil recruitment in IAV-*Af* co-infected mice was transient, followed by a subsequent increase in neutrophil levels in co-infected lungs (48 hours). RNA sequencing (RNAseq) analysis of neutrophils from IAV-*Af* co-infected lungs (48 hours) exhibited the enrichment of cellular pathways regulating inflammatory response and phagocytosis. Despite the up-regulation of inflammatory and phagocytosis-related pathways in neutrophils, the host response from co-infected mice suppressed neutrophil-mediated conidial killing. However, the increased *Af* invasion at 24 hours post co-infection, despite similar fungal loads in both groups (*Af* vs. IAV-*Af*), suggests that IAV-induced pathological lung inflammation and vascular damage enhance *Af* invasiveness during the early phase of co-infection, while impaired neutrophil fungicidal response and exacerbated lung damage likely contribute to sustained *Af* invasiveness and fatal IPA pathogenesis in the later phase of co-infection.

## RESULTS

### IAV promotes severe *Af* infection (IPA) in a murine co-infection model

**Fig 1:**
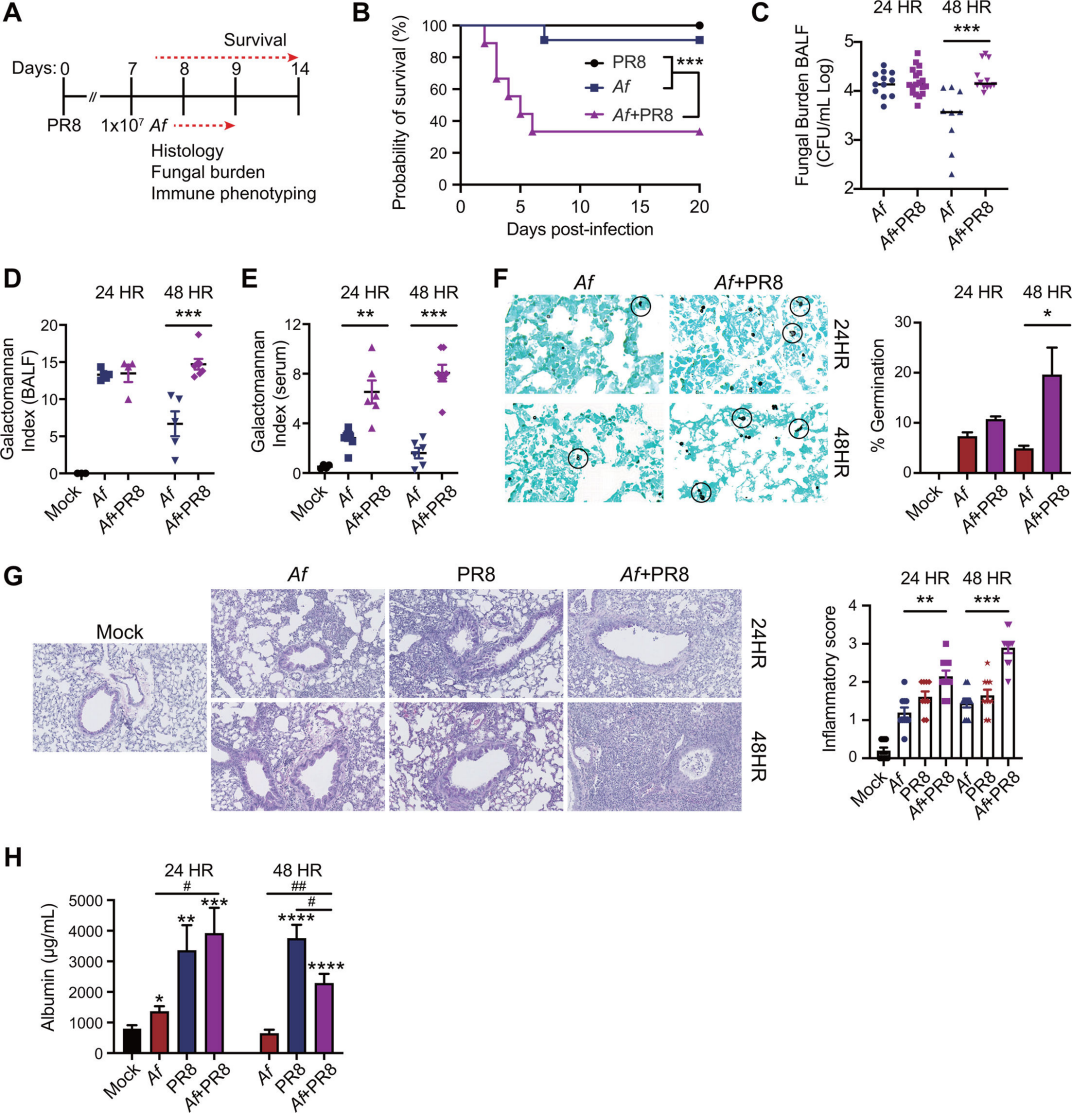
A murine model of IAV and *Af* co-infection. Equal proportion of age-matched (6-8 weeks) male and female C57BL/6J mice were intranasally infected with 250 PFUs of IAV on day 0. At day 7, mice were infected with 1x10^7^ *Aspergillus fumigatus* resting conidia. Mice were euthanized at 24 and 48 hours post-single *Af* infection or co-infection for fungal burden, histological analysis, and immune phenotyping. (A) Experimental outline. (B) Survival curve of single IAV, *Af,* and IAV-*Af* co-infected mice. n = 10/group of two independent experiments. (C) Fungal burden in bronchoalveolar lavage fluid (BALF) expressed as Log CFU/mL. (D) Detection of galactomannan antigen in BALF. (E) Detection of galactomannan antigen in serum. (F) Percent of germinating conidia, expressed as % germination, quantitated using silver stain for observation of swollen and germinating conidia (n=5 mice/group of one independent experiment). (G) H&E stain of lung tissue with inflammatory score (0 indicating little to no leukocyte infiltration and 4 indicating severe leukocyte infiltration). (H) Albumin concentration in BALF. n = 5/group of one independent experiment. Data were representative of two independent experiments, unless mentioned otherwise. Data were shown as mean ± SEM and analyzed by Mantel-Cox log-rank test (B) or one-Way ANOVA with Tukey’s post-hoc analysis (C through F). *p ≤ 0.05, **p ≤ 0.01, ***p ≤ 0.001 and ****p ≤ 0.0001. In G, * denotes differences from control (mock), and ^#^ denotes differences between groups.

To investigate the association of IAV with secondary *Af* infection, we infected age-matched male and female C57BL/6 mice with a sublethal dose of 250 PFU of IAV (A/PR/8/34 or PR8). Seven days later, the IAV-infected mice were co-infected with 1 × 10^7^ *Af* resting conidia (strain NIH 5233) (Fig. 1A). Co-infection with 1 × 10^7^ conidia resulted in approximately 70% mortality in co-infected mice, compared to nearly sublethal outcomes with single *Af* or IAV infections (Fig. 1B). By 48 hours post co-infection, the co-infected mice exhibited a significantly higher fungal burden and conidial germination compared to a single *Af* infection, as determined by colony forming unit (CFU), galactomannan antigen index in bronchoalveolar lavage fluid (BALF) and serum, and lung silver staining (Fig. 1C through F). No difference in fungal burden or conidial germination was observed at the 24 hour time point between single *Af* and co-infected lungs (Fig. 1C through F). However, despite similar fungal burden and conidial germination between single *Af* and co-infected mice, a significant increase in serum galactomannan antigen was detected in co-infected mice at 24 hours, with a further increase observed at 48 hours post co-infection (Fig. 1C through F), suggesting the development of IPA in co-infected mice. The serum galactomannan antigen index is a reliable marker for diagnosing IPA (8, 9). Compared to single IAV or *Af* infections, co-infected mice exhibited a synergistic increase in lung inflammation at 24 and 48 hours post co-infection (Fig. 1G). However, IAV infection alone caused significant vascular injury, as indicated by increased albumin levels in BALF, while co-infection with *Af* did not further exacerbate vascular leakage (Fig. 1H). Interestingly, despite higher lung inflammatory scores at 48 hours post co-infection, albumin levels were reduced. Further studies are needed to determine the factors influencing vascular permeability during the later stage of co-infection.

### IAV suppresses lung neutrophil recruitment during the initial phase of co-infection, followed by a second wave of robust neutrophil responses

**Fig 2:**
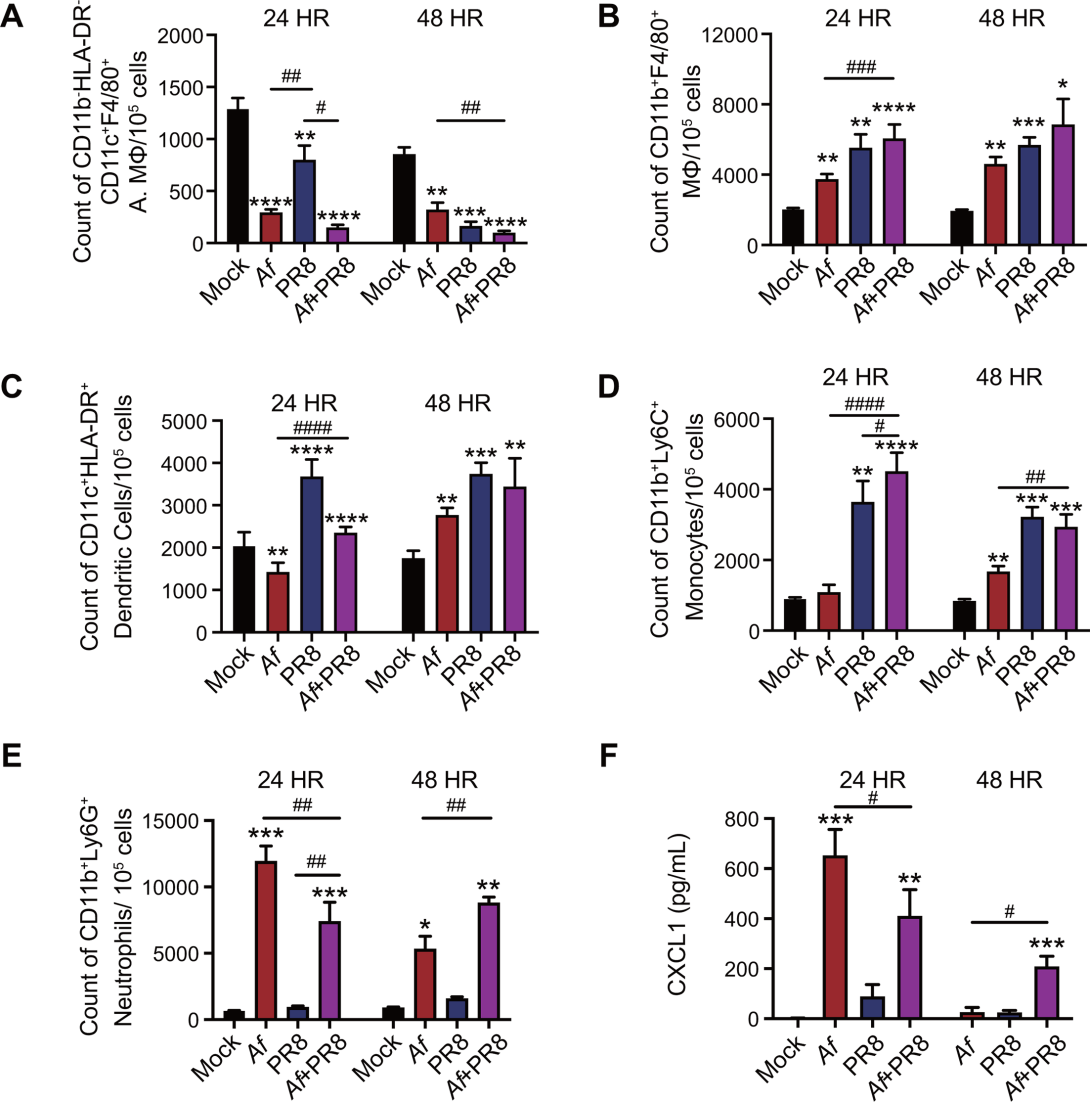
Host response to IAV-*Af* co-infection. Equal proportion of age-matched (6–8 weeks) male and female C57BL/6J mice were intranasally infected with 250 PFU of IAV on day 0. At day 7, mice were infected with 1 × 10^7^ *Aspergillus fumigatus* (A*f*) resting conidia. Mice were euthanized at 24 and 48 hours post-single *Af* infection or co-infection for lung immune analysis. (**A**) Flow cytometry count of lung alveolar macrophages (A. MΦ, CD11b^−^HLA-DR^−^CD11c^+^F4/80^+^) at 24 and 48 hours. (**B**) Count of lung macrophages (MΦ, CD11b^+^F4/80^+^). (**C**) Count of lung dendritic cells (CD11c^+^HLA-DR^+^) (**D**) Count of lung monocytes (CD11b^+^Ly6C^+^). (**E**) Count of lung neutrophils (CD11b^+^Ly6G^+^). (**F**) CXCL1 chemokine concentrations in lung homogenates. Data were representative of two independent experiments. Data were shown as mean ± SEM. Statistics were performed using one-way ANOVA with Tukey's post-hoc analysis; *P ≤ 0.05, **P ≤ 0.01, ***P ≤ 0.001, and ****P ≤ 0.0001. * denotes differences from control (mock), and ^#^ denotes differences between groups.

We performed flow cytometry to investigate the innate immune cells in the lungs of single (IAV, *Af*) and IAV-*Af* co-infected mice. Our data indicate that, in comparison to mock and *Af* infection, IAV-infected lungs exhibited markedly increased frequencies of inflammatory monocytes, dendritic cells, and macrophages (MΦ) (Fig. 2A through D). However, both *Af* and IAV single infections led to a significant reduction in alveolar macrophages (A. MΦ). Co-infection with *Af* did not significantly alter the frequencies of monocytes, dendritic cells, or macrophages at 24 or 48 hours compared to IAV alone, indicating that IAV is the main driver of these responses during co-infection. No difference in lung neutrophil levels was observed between mock and IAV infection (Fig. 2E). In contrast, *Af* infection resulted in robust recruitment of neutrophils, which was associated with fungal clearance in the lung. IAV inhibited early neutrophil recruitment induced by *Af* at 24 hours (Fig. 2E), correlating with decreased levels of CXCL1 (Fig. 2F). Despite the reduced neutrophil levels at 24 hours post co-infection, there was no difference in fungal burden or conidial germination between mice infected with *Af* alone and those co-infected with IAV and *Af* (Fig. 1C through F). Paradoxically, IAV-*Af* co-infected mice had significantly higher neutrophil levels at 48 hour time points, correlating with higher fungal burden in the lung. These data suggest that although IAV host response initially suppressed neutrophil recruitment during IAV-*Af* co-infection, no long-term defects in neutrophil recruitment were observed in co-infected lungs.

### RNAseq analysis reveals the heightened pro-inflammatory phenotype of neutrophils in IAV-*Af* co-infected lungs

**Fig 3:**
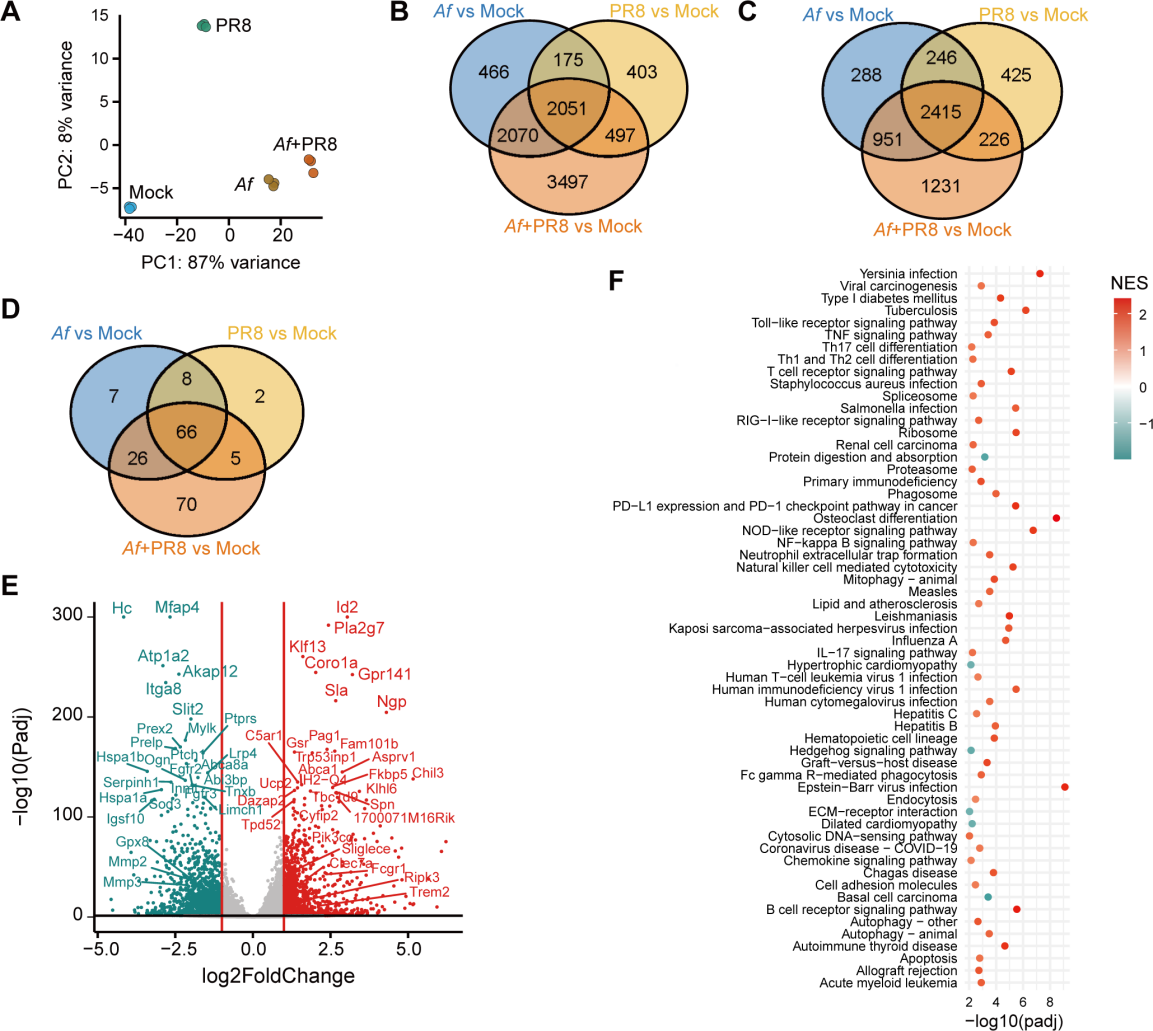
Neutrophil gene expression profiles. Equal proportion of age-matched (6–8 weeks) male and female C57BL/6J mice were intranasally infected with 250 PFU of IAV on day 0. At day 7, mice were infected with 1 × 10^7^ *Aspergillus fumigatus* (A*f*) resting conidia. Mice were euthanized at 48 hours post-single *Af* infection or co-infection, BALF was collected and pooled within groups, and neutrophils were sorted for RNAseq analysis, *n* = 3 for each group. (**A**) Principal component analysis (PCA) is based on the 500 most variant expressed genes across samples. Color stands for the groups. (**B**) Venn diagram showing the overlapping and unique differentially expressed genes (DEGs) from all three comparisons. DEGs were identified with adjusted *P*-value < 0.05 and an absolute log2 fold change > 1. (**C**) Venn diagram showing the overlapping and unique significant enriched Gene Ontology biological processes from all three comparisons. Significant GO biological processes were identified with adjusted *P*-value < 0.05. (**D**) Venn diagram showing the overlapping and unique significant enriched KEGG pathways from all three comparisons. Significant KEGG pathways were identified with adjusted *P*-value < 0.05. (**E**) Volcano plot showing the global transcriptional changes in IAV-*Af* versus *Af* infection group. Each dot represents one gene. The gray dots represent no significant DEGs between IAV-*Af* and *Af* infection groups, the blue dots represent down-regulated genes, and the red dots represent up-regulated genes. (**F**) Dotplot showing the significant pathways identified from Gene Set Enrichment Analysis (GSEA) with KEGG pathway between IAV-*Af* versus *Af* groups. Significant KEGG pathways were identified with adjusted *P*-value < 0.01. The color stands for up-regulated (red) or down-regulated (blue) in the IAV-*Af* group.

Neutrophils function as a double-edged sword: while they are crucial for clearing bacterial or fungal pathogens in the lung, excessive neutrophilia can contribute to lung pathology and promote microbial invasion of the bloodstream (10–12). In our IAV-*Af* co-infection model, despite higher neutrophil levels at 48 hours compared to the single *Af* infection (Fig. 2E), we observed excessive lung pathology, increased conidial germination, and fungal invasion of the bloodstream (serum galactomannan) (Fig. 1C through E). We speculated that, while inflammatory monocytes and macrophages are the primary drivers of lung tissue damage in IAV infection, neutrophils contribute to tissue pathology during the later phase of co-infection (48 hours). To investigate this, we performed an unbiased RNA sequencing (RNAseq) analysis to compare the inflammatory responses of neutrophils between single *Af* and IAV-*Af* co-infected lungs, 48 hours after establishing *Af* co-infection (Fig. S1 to S4). The principal component analysis (PCA) plot revealed clear separation among all groups (mock, *Af*, IAV, and IAV-*Af*) (Fig. 3A), with the highest number of differentially expressed genes (DEGs) observed in both *Af* vs. mock and IAV-*Af* vs. mock comparisons (Fig. 3B; Fig. S1 to S3). Furthermore, Gene Ontology (GO) enrichment analysis revealed that 951 significantly enriched biological processes were shared between the *Af* vs. mock and IAV-*Af* vs. mock comparisons (Fig. 3C). KEGG functional enrichment analysis identified 66 significant pathways shared among all groups, with the highest number, 70 pathways, uniquely identified in the IAV-*Af* vs. mock comparison (Fig. 3D). Notably, in the IAV-*Af* co-infected group, a significant number of pro-inflammatory and apoptotic genes were up-regulated compared to the *Af* group (Fig. 3E). Additionally, Gene Set Enrichment Analysis (GSEA) showed that neutrophils from co-infected mice, compared to those from *Af*-infected mice, demonstrated significant enrichment of pathways related to inflammation, cell death, neutrophil extracellular trap formation, and cellular cytotoxicity (Fig. 3F). These findings suggest that hyperinflammatory neutrophil responses during the later phase of IAV-*Af* co-infection are potentially linked to pathologic lung inflammation and tissue damage.

### IAV host response enhances neutrophil phagocytosis pathways but suppresses fungal killing responses

**Fig 4:**
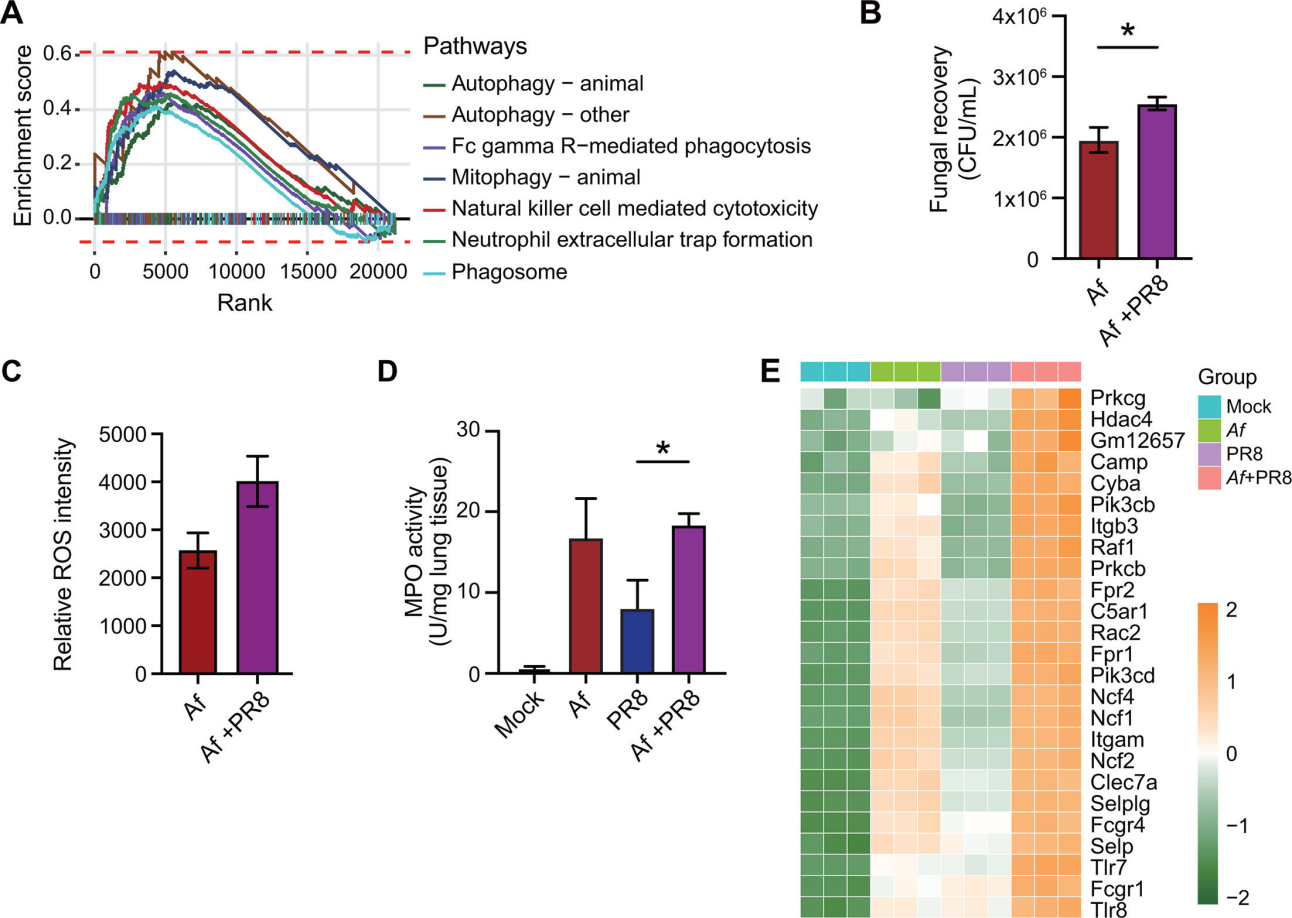
*In vitro* neutrophil phagocytosis. Equal proportion of age-matched (6–8 weeks) male and female C57BL/6J mice were intranasally infected with 250 PFU of IAV on day 0. At day 7, mice were infected with 1 × 10^7^ *Aspergillus fumigatus* (*Af*) resting conidia. Mice were euthanized at 48 hours post-single *Af* infection or co-infection, BALF was collected and pooled within groups, and neutrophils were sorted for RNAseq analysis. (**A**) Gene Set Enrichment line plot shows significantly enriched KEGG pathways between IAV-*Af* versus *Af* groups with the enrichment score indicated. (**B**) Fungal recovery as determined by CFU from neutrophils treated with pathogen-purified BALF from *Af* or IAV-*Af* co-infected mice. (**C**) Relative reactive oxygen species (ROS) fluorescence intensities by DCFDA assay. (**D**) Myeloperoxidase (MPO) activity is measured in lung tissue and represented as a unit per milligram of tissue. (**E**) Heatmap shows the expression profiling of significant genes from “Neutrophil extracellular trap formation” pathway. Data were shown as mean ± SEM. Statistics were performed using Student's *t*-test (**B and D**) or one-way ANOVA with Tukey's post-hoc analysis (**C**). *P ≤ 0.05.

While IAV-induced tissue damage alone promotes bacterial growth and invasion (11, 13–16), it remains unclear whether impaired neutrophil phagocytic and microbicidal functions due to IAV directly contribute to conidial germination and fungal invasion of damaged lung vasculature in IAV-Af co-infected mice, as most published studies lack an unbiased, in-depth characterization of neutrophil responses. To address this, we first analyzed neutrophil RNAseq data to investigate the differences in phagocytic responses between *Af*-infected and IAV-*Af* co-infected mice at 48 hours post-single *Af* or IAV-*Af* co-infection. The RNAseq findings reveal that, compared to *Af* single infection, several cellular pathways involved in phagocytic responses, such as autophagy, Fcγ receptor-mediated phagocytosis, mitophagy, phagosome, and NET formation, were significantly up-regulated in neutrophils from IAV-*Af* co-infected lungs (Fig. 3F and 4A). DEGs related to NET formation were up-regulated in IAV-*Af* co-infection compared to single *Af i*nfection, as well as in comparison to Mock or IAV infection (Fig. 4E). Next, to determine whether the host response from co-infected lungs enhances neutrophil-mediated *Af* killing similarly to its effect on phagocytic function, we treated peritoneal neutrophils with pathogen-free BALF from *Af* and IAV-*Af* co-infected mice (48 hours post-infection) and evaluated their impact on *Af* intracellular killing *in vitro*. Our data show that neutrophils treated with BALF from co-infected lungs (compared with *Af*) had higher fungal recovery based on CFU recovered from neutrophil lysates (Fig. 4B). Interestingly, despite defects in fungal killing, the neutrophils from co-infected lungs expressed higher levels of reactive oxygen species (ROS) and myeloperoxidase (MPO) (Fig. 4C and D), suggesting that fungal killing by neutrophils during co-infection occurs independently of ROS and MPO. These findings suggest that, while IAV host response limits neutrophil recruitment and fungicidal activity, it enhances neutrophil phagocytosis and inflammatory functions, potentially contributing to increased lung vascular inflammation and insufficient fungal control. However, the increased fungal invasion observed at 24 hours post co-infection, despite similar lung fungal loads in both groups (*Af* vs. IAV-*Af*), suggests that pathological lung inflammation and vascular damage may play a more profound role in driving the development of IPA in the context of IAV co-infection.

## DISCUSSION

Influenza virus presents a growing and significant risk for secondary *Af* infections, which can progress into life-threatening invasive pulmonary aspergillosis. Unlike the well-established models of influenza-bacterial co-infections that mimic the natural pathogenesis of secondary bacterial infections, the limited IAV-*Af* animal co-infection models hamper further study of the association between IAV and secondary *Af* infections. In this study, we developed an IAV-*Af* co-infection model to investigate the role of IAV host response in influenza-associated pulmonary aspergillosis. Our findings add to a growing body of literature highlighting the multifactorial pathogenesis of influenza-associated pulmonary aspergillosis, including early suppression of neutrophil recruitment, pathologic lung inflammation, and impaired neutrophil fungicidal activity, which may collectively contribute to the development and progression of IPA during IAV infection.

Our data show that IAV infection attenuates neutrophil recruitment during the early phase of *Af* co-infection. While Tobin et al. reported IAV-mediated suppression of neutrophil recruitment via CXCL1 (13), our data extend these findings by demonstrating that this suppression is transient and followed by a hyperinflammatory neutrophil response. Importantly, using a different *Af* strain, we observed similar findings on early neutrophil recruitment defects, indicating that IAV-mediated susceptibility to IPA is independent of fungal strain background. Despite reduced neutrophil recruitment at 24 hours post co-infection, fungal burden remained comparable between *Af*-infected and IAV-*Af* co-infected lungs. Notably, co-infected mice exhibited increased *Af* tissue invasiveness even at this early time point, suggesting that non-neutrophil factors likely contribute to fungal progression during the initial phase of co-infection. Given the crucial role of lung vascular injury in promoting the hematogenous dissemination of *Af*, our findings indicate that IAV-induced lung inflammation and vascular barrier disruption likely facilitate early fungal invasion and systemic spread, as supported by the detection of galactomannan antigen in serum. The observation that fungal invasiveness is enhanced despite equivalent fungal load further implicates IAV-mediated epithelial and vascular injuries as key determinants of early susceptibility. This aligns with findings by Jamieson et al., who showed that mortality in IAV-bacterial co-infections can result from failure to tolerate tissue damage rather than microbial burden. Epithelial barrier disruption has been identified as a central driver of susceptibility to secondary bacterial infections following influenza (14, 15), and similarly, prior studies have implicated lung barrier dysfunction as a key factor in the pathogenesis of invasive pulmonary aspergillosis (16).

In contrast to the early phase, the later stage of co-infection (48 h) was marked by elevated neutrophil accumulation, which correlated with increased lung inflammation, higher *Af* burden, and greater tissue invasiveness compared to *Af*-only infection. While most existing IAV-*Af* models focus on late-stage pathology, our model captures both 24 and 48 hour post-*Af* time points, uniquely delineating a progression from early epithelial and vascular barrier disruption to a delayed, hyperinflammatory neutrophil response. To investigate whether impaired neutrophil antifungal responses contribute to fungal outgrowth in IAV-*Af* co-infected mice, we performed RNAseq on lung neutrophils 48 hours post co-infection and compared their profiles to those from *Af*-only infected mice. Neutrophils from co-infected lungs exhibited a hyperinflammatory transcriptomic signature with up-regulation of pathways related to phagocytosis, including phagosome formation, autophagy, mitophagy, NET formation, FcγR-mediated phagocytosis, and oxidative response. Liu et al. previously demonstrated that IAV impairs antifungal defense by disrupting phagocyte function at multiple levels, including defects in phagolysosomal maturation in neutrophils and impaired ROS production and LAP activation in macrophages, leading to defective killing of *Af* conidia despite preserved uptake (17). We found that neutrophils from IAV-*Af* co-infected mice exhibit a hyperinflammatory transcriptomic profile with increased expression of phagocytic and ROS-related genes. Yet, functionally, these neutrophils exhibited impaired fungicidal activity *in vitro* when exposed to BAL fluid from co-infected mice. These findings support a model in which IAV-induced inflammation enhances phagocytic signaling without restoring fungicidal capacity, suggesting that inhibitory cues in the post-IAV lung environment actively decouple effector activation from fungal clearance. Notably, while Liu et al. focused on direct cell-intrinsic defects, our study reveals that the inflammatory context, including soluble mediators in BAL, may further suppress neutrophil killing functions, contributing to persistent fungal invasion. This supports a broader concept in which IAV-induced dysregulation of both cellular programs and microenvironmental signals undermines innate antifungal immunity. These findings also support prior studies in IAV-bacterial co-infection models, where excessive neutrophilia was linked to tissue injury rather than improved microbial clearance (18, 19).

Impaired phagocytic response is a hallmark of susceptibility to pulmonary aspergillosis, particularly in immunocompromised hosts (20). In both human and murine models, neutrophils and inflammatory monocytes are critical for restricting *Af* germination and invasion (21, 22). However, in IAV-infected lungs, the immune landscape is dominated by excessive inflammation rather than immune suppression. Prior studies have shown that monocytes and macrophages contribute to IAV-driven lung injury (23, 24), and recent work by Seldeslachts et al. demonstrated that excessive IFN-γ impairs macrophage fungal killing by disrupting LC3-associated phagocytosis (25). Our findings build on this by showing that IAV-induced vascular and epithelial damage facilitates early Af invasion, even in the absence of increased fungal burden. At later stages, neutrophils in co-infected lungs exhibit hyperinflammatory and phagocytic signatures but fail to control fungal growth, revealing a functional decoupling between neutrophil activation and fungal clearance. Together, these findings demonstrate that IAV-induced tissue injury and dysregulated innate immune responses synergize to promote fungal pathogenesis, shifting the balance from protective to pathogenic inflammation. While our study provides important mechanistic insights, it is limited by the use of a single *Af* strain and murine background, which may not fully capture the heterogeneity of human IPA. Additionally, as our RNAseq analysis was performed at a single 48 hour time point, further temporal profiling is needed to define how neutrophil function evolves across the course of co-infection. Such insights will be essential for identifying therapeutic windows to modulate inflammation without compromising pathogen control.

## MATERIALS AND METHODS

### Mouse model of IAV-*Af* co-infection

Wild-type (WT) C57BL/6J mice (6–8 weeks old) were purchased from Jackson Laboratory (Bar Harbor, ME) and bred in-house. Age- and sex-matched mice were used for experiments and received food and water *ad libitum*. Influenza A Virus (H1N1 A/Puerto Rico/8/1934 or PR8) was purchased from Charles River, Norwich, CT. *Aspergillus fumigatus* (*Af*) strain (NIH 5233) was purchased from ATCC and prepared according to the manufacturer's recommendation. For experiments, *Af* was grown in flasks on Sabouraud dextrose agar (SDA) for 7 days at 35°C with 5% CO_2_, as previously mentioned (26). On day 8, the mature conidia were suspended in a solution of sterile phosphate-buffered saline (PBS) containing 0.05% Tween 80. The solution containing *Af* was filtered through sterile gauze to remove hyphae, and the number of conidia was counted using a hemocytometer. For *Af* infection, mice were anesthetized (4% vol/vol isoflurane/oxygen) and intranasally administered with 1 × 10^7^ resting conidia in 50 µL of PBS. For IAV infection, mice were similarly anesthetized and intranasally administered 250 PFU of PR8 in 70 µL PBS. For the IAV-*Af* co-infection model, mice were infected with IAV as previously mentioned. On day 7 post-IAV infection, mice were infected with *Af* (as mentioned above). For experiments, mice were euthanized 24 and 48 hours post-*Af* infection. Bronchiolar lavage (BAL) fluid was collected via the intratracheal administration of 1 mL of ice-cold PBS and centrifuged at 800 × g for 10 min, and the supernatant was preserved at −20°C for chemokine analysis. The lungs were perfused with sterile PBS, aseptically removed, and processed accordingly for downstream applications.

### Flow cytometry

For lung single-cell suspensions, lungs were aseptically excised and digested with 5 mL of media containing collagenase (25 U/mL) and DNase (0.5 mg/mL) for 30 min prior to passing through 70 µm cell strainers. Red blood cells (RBCs) were lysed using ammonium–potassium–chloride (ACK) lysis buffer (Life Technologies, Carlsbad, CA) and washed twice in PBS supplemented with FBS. Cells were counted using a hemocytometer, and 1 × 10^6^ cells were surface stained in FACS buffer containing antibodies against CD11b (M1/70), Ly6C (HK1.4), Ly6G (1A8), IA/I-E (M5/114.15.2), CD11c (N418), and F4/80 (BM8) (BioLegend, San Diego, CA). We acquired 100,000 events/samples on a BD FACSymphony A3 flow cytometer, and the data were analyzed using FlowJo (BD) according to the gating strategy in Fig. S1.

### CXCL1 ELISA

The lungs were homogenized in T-PER tissue protein extraction reagent (#78510, Thermo Fisher Scientific), centrifuged at 10,000 × g for 10 min, and the supernatants were collected. CXCL1 chemokine concentrations in lung samples were measured using a murine-specific CXCL1 ELISA kit (Thermo Fisher Scientific) according to the manufacturer's instructions. Briefly, lung homogenate supernatant was incubated on a pre-coated CXCL1 sandwich ELISA plate with the provided standard. The plate was measured at 450 nm with a Synergy HT (Bio-Teck, Vermont, CA), and the total CXCL1 per milliliter of lung homogenate was calculated by generating the standard curve provided.

### Histopathology

The left lobe of the lung was perfused with PBS and fixed in 10% neutral buffered (pH 7.4) formalin for 24 h at room temperature. Then, the lung tissues were embedded in paraffin and sliced into 5 µm sections to reveal the main bronchus. Slides were stained with hematoxylin and eosin (H&E) to assess inflammation and silver stain (Modified GMS, Sigma-Aldrich) to assess germination. For inflammatory scoring, all slides were coded and evaluated by three pathologists blinded to the experimental groups. Each tissue section was scored based on a scale of 0–4 with increments of 0.5, with 0 as no inflammation and 4 as the highest degree of immune cell infiltration (27). Representative histological images were acquired using a NanoZoomer 2.0-HT Brightfield Fluorescence Slide Scanning System (Hamamatsu Photonics, Japan) and analyzed using NDP.view2 Viewing Software (Hamamatsu). Fungal germination was quantified as described previously (28). Briefly, total and germinating conidia were counted in 50 random fields of view (400 × magnification) on each slide. Germinating conidia were defined as swollen conidia (two times the size of normal conidia) and conidia that have started producing a germ tube. Data are presented as % germination or ratio of germinating conidia (swollen and/or germ tube formation) to total conidia in a field of view.

### RNAseq

Neutrophils were magnetically sorted from pooled BALF from mock, single (*Af*, IAV), and IAV-*Af* co-infected mice (*n* = 3/group) at 48 hours after single/co-infection, by using MACS anti-Ly6G microbead kit (#130-092-332, Miltenyi Biotec) according to manufacturer's instructions. RNA extraction was performed using the Qiagen RNA extraction kit. Libraries were prepared with a NEBNext Ultra II RNA-Seq library kit following the manufacturer's instructions as described earlier (23). Quality control for each sample was performed to determine the RNA quantity before RNAseq. Samples were run on one NovaSeq 6000 lane. Raw reads with low-quality (Q < 30) reads and sequencing adapters were removed, and the remaining clean reads were mapped to the mouse reference genome mm10 (GRCm38) with HISAT2 (29). FeatureCount was used to summarize the reads mapped to mouse genes (30). DESeq2 was used to identify differentially expressed genes (DEGs) with an adjusted *P*-value < 0.05 and an absolute log2 fold change > 1 (31). Enriched Gene Ontology (GO) biological processes and Kyoto Encyclopedia of Genes and Genomes (KEGG) pathways were identified by richR (https://github.com/hurlab/richR), with an adjusted *P*-value < 0.05 as the significance cut-off. Additionally, Gene Set Enrichment Analysis (GSEA) with KEGG pathways was performed to identify pathways that show significant differences and the directionality of changes between biological samples, with an adjusted *P*-value < 0.01 using richR.

### Fungal burden and galactomannan antigen detection assay

To determine fungal burden, we serially diluted BAL fluid in sterile PBS and plated it onto Petri plates containing SDA medium. Plates were incubated at 35°C overnight, and CFU were enumerated and expressed as CFU/mL of BAL fluid. The Platelia *Aspergillus* Ag (Bio-Rad) was used for the detection of *galactomannan* antigen in serum and BAL fluid. The assay was performed according to the manufacturer's recommendation, and the plate was measured at 450 nm with a Synergy HT (Bio-Tek, Vermont, CA). The data are reported as calculated index values.

### Albumin ELISA

Albumin concentrations in BAL fluid samples were measured using a murine-specific albumin ELISA kit (ALPCO Diagnostics, Salem, NH). The assay was performed according to the manufacturer's instructions. The plate was measured at 450 nm with a Synergy HT (Bio-Teck, Vermont, CA), and total BALF albumin was calculated by generating a standard curve.

### Neutrophil phagocytosis assay

Peritoneal neutrophils were isolated from naïve C57BL/6 mice, as previously described (32). Neutrophils (1 × 10^6^/mL) were plated in 96-well plates and pulsed with 100 µL of pathogen-purified BALF from *Af* or IAV-*Af* co-infected mice 48 hours after a single *Af* co-infection. After 1 hour of BALF treatment, neutrophils were infected with resting *Af* conidia for 6 hours at an MOI of 1. The cells were washed and lysed, and serial dilutions of cell lysates were plated on SDA media overnight at 35°C before colonies were counted.

### Myeloperoxidase (MPO) Assay

MPO assay was performed as described earlier (33). Briefly, lung tissue weight was recorded, and hexadecyltrimethylammonium bromide (HTAB) buffer was added in the ratio of 12.5 mg/mL. Tissue samples were homogenized for 4 min at 30 Hz. Homogenized solutions were centrifuged for 6 min (13,400 × g, 4°C), and supernatants were used to quantify MPO activity. For the MPO activity measurement, 7 µL supernatants were added to the 96-well plate in triplicates, followed by the addition of 200 µL of the o-dianisidine mixture containing H_2_O_2_. The absorbance was measured immediately at 450 nm using a spectrophotometer. The three readings at 30 second intervals were recorded, and MPO activity was calculated in units (U) of MPO/mg tissue for each sample.

### Reactive oxygen species (ROS) detection

From the BALF of each experimental group of mice, the neutrophils were magnetically sorted 48 hours after single/co-infection. The neutrophil's ROS production was assessed using the DCFDA/H2DCFDA-Cellular ROS Assay Kit from Abcam (Cat. No. ab113851). The assay was performed as per the manufacturer's instructions. In brief, for each experimental condition, isolated neutrophils were incubated at 1.5 × 10^5^ cells per milliliter with the DCFDA probe at 37°C for 30 minutes in the dark, followed by a wash with 1× buffer. Neutrophils were resuspended in 1× Buffer at the concentration 1 × 10^6^ cells/mL and seeded in a dark, clear bottom 96-well microplate with 100,000 stained cells/well. The plate measurement was taken immediately on a fluorescence plate reader at Ex/Em = 485/535 nm in endpoint mode. The data analysis was performed with GraphPad Prism 10 software.

## Data Availability

RNA sequencing data is accessible at accession no. GSE276049. All other data generated or analyzed during this study are within the manuscript.

## SUPPLEMENTAL MATERIAL

Fig. S1 iai.00234-25-s0001.docx Flow cytometry gating strategy of immune cells.

Table S1 iai.00234-25-s0002.xlsx Differential gene expression analysis results from DESeq2 between *Af* versus Mock.

Table S2 iai.00234-25-s0003.xlsx Differential gene expression analysis results from DESeq2 between IAV versus Mock.

Table S3 iai.00234-25-s0004.xlsx Differential gene expression analysis results from DESeq2 between IAV-*Af* versus Mock.

Table S4 iai.00234-25-s0005.xlsx Differential gene expression analysis results from DESeq2 between IAV-*Af* versus *Af*.

ASM does not own the copyrights to Supplemental Material that may be linked to, or accessed through, an article. The authors have granted ASM a non-exclusive, world-wide license to publish the Supplemental Material files. Please contact the corresponding author directly for reuse.
