# Supplementary material for: Loss of pulmonary tissue protection and neutrophil microbicidal defects promote severe Aspergillus fumigatus infection during influenza A virus infection
Source: Infect Immun. 2025 Aug 11;93(9):e00234-25. doi: 10.1128/iai.00234-25 (PMC12418741; doi:10.1128/iai.00234-25)
Supplement: Fig. S1 — Flow cytometry gating strategy of immune cells. [file iai.00234-25-s0001.docx]

**SUPPLEMENTARY FIGURES & FILES**

**
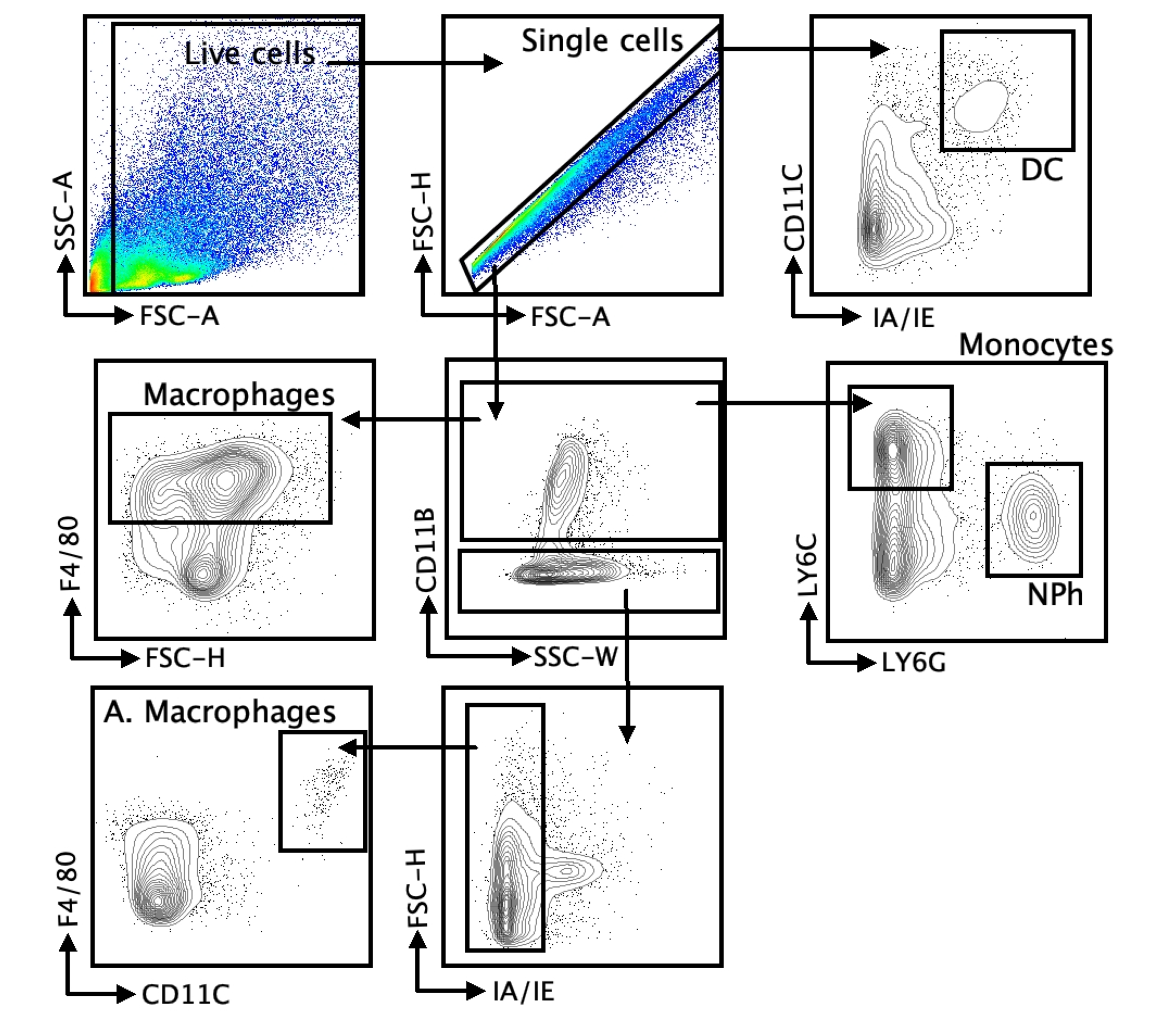
**

**FIG S1. Flow cytometry gating strategy of immune cells.**

Lung single cells were isolated and prepared as described in the methods. Prepared cells were run on a BD FACSymphony flow cytometer immediately following staining, and data were analyzed using FlowJo. Live cells were gated using forward scatter and side scatter to exclude debris. Single cells were gated using forward scatter area versus forward scatter height. Dendritic cells (DC) were analyzed using CD11C versus IA/IE (double-positive). CD11b^+^ cells were gated to analyze CD11b^+^Ly6C^+^ monocytes, CD11b^+^Ly6G^+^ neutrophils (NPh), and CD11b^+^F4/80^+^ macrophages. The CD11b^-^ population was gated to identify IA/IE-F4/80^+^CD11C^+^ alveolar macrophages.
